# Burnout and perceived health in medical residents after the COVID-19 pandemic: a single-center cross-sectional study

**DOI:** 10.3389/fpsyt.2026.1659089

**Published:** 2026-06-19

**Authors:** Victoria Olivé, Ricard Navinés, Laura Pujol, Klaus Langohr, Eduard Vieta, Rocio Martin-Santos

**Affiliations:** 1 Department of Medicine, Facultat de Medicina i Ciències de la Salut, Universitat de Barcelona (UB), Barcelona, Spain; 2 Department of Occupational Health and Prevention, Hospital Clinic, UB, Barcelona, Spain; 3 Program of Mental Health of the Hospital Workers. Department of Psychiatry and Psychology, Hospital Clinic, Institut d´Investigacions Biomëdiques August Pi i Sunyer (IDIBAPS), and Centro de Investigación Biomédica en Red en Salud Mental (CIBERSAM), Barcelona, Spain; 4 Department of Statistics and Operations Research, Biostatistics and Bioinformatics Research Group, and Institute for Research and Innovation in Health (IRIS), Universitat Politècnica de Catalunya - BarcelonaTech, Barcelona, Spain; 5 Integrative Pharmacology and Systems Neuroscience Research Group, Hospital del Mar Research Institute, Barcelona, Spain

**Keywords:** burnout residents, COVID-19 pandemic, FPSICO-4.0 questionnaire, overall job satisfaction, psychosocial risk, SF-36 questionnaire

## Abstract

**Introduction:**

Residents have a high risk of burnout during the training period. The COVID-19 pandemic has had a great impact on work conditions and health of medical residents. We therefore estimated the prevalence of post-pandemic burnout and perceived health, and their relationship with psychosocial factors, stress profile, and job satisfaction, as well as the pre-post pandemic changes.

**Methods:**

We carried out a retrospective cross-sectional, online, anonymous survey using the validated FPSICO-4.0 questionnaire in a teaching hospital after the COVID-19 pandemic (2023). We also studied the pre-post changes from an independent pre-pandemic online survey (2018) with the same tool.

**Results:**

One hundred ninety-seven residents, 64% females, answered the questionnaire (43.5%); 28% had post-pandemic burnout without gender differences, being higher in surgical specialties (p=0.017) and along residency years (p=0.041). Role performance, behavior stress, job satisfaction and residency years were associated with an increase in odds of burnout (p<0.05). Supervision, conflict of role/cognitive stress explained 35% of the variation in perceived general health (adjusted-R2 = 0.356); temporary autonomy, supervision, information/training/promotion, behavior/somatic stress, and job satisfaction did 68% of mental health (adjusted-R2 = 0.683); and take decisions, conflict of role, behavior/somatic/cognitive stress, and male gender did 62% of vitality (adjusted-R2 = 0.624). Pre/post pandemic mean differences, adjusted by gender and residency year, showed deterioration of variety sense of work (p<0.001), effort of attention (p=0.026), behavior stress (p=0.022), and improvement of temporary autonomy (p=0.045).

**Conclusions:**

The identification after pandemic of high prevalence of burnout, stress levels and certain psychosocial risk factors (role performance and supervision), highlights the need of specific health promotion plan.

## 1 Introduction

Healthcare professionals are a working population group highly exposed to stress, being medical residents particularly vulnerable (1, 2). Residency training is a challenging period, where while learning specialist skills, a multitude of new stressors are faced. These stressors may be related to the medical profession itself (workload, dealing with serious illnesses, new technologies…) or to other non-medical factors (moving place, new relationships…).

The biological activation that defines the body stress response is accompanied by unpleasant emotions (anxiety, sadness…) and by physiological modifications of the adaptive subsystems (autonomic and neuroendocrine activation and immune and behavior inhibition) (3). When prolonged in time and intensity, this response becomes anti-homeostatic and leads to a repercussion on physical and mental health (i.e. metabolic syndrome, burnout, depression) (4).

Burnout is recognized as an occupational syndrome characterized by emotional and cognitive disturbances, typically presenting as emotional exhaustion, depersonalization or cynicism, and a reduced sense of personal achievement (5). This condition arises from continuous and overwhelming work-related stress and demands (4, 6). Prolonged exposure to uncontrollable stress has marked deleterious effects on the prefrontal cortex, a brain region responsible for key cognitive functions essential to physicians, such as abstract thinking, complex decision-making, self-reflection, and sustained effort in the face of adversity (7). By definition, occupational burnout is primarily precipitated by characteristics of the work environment rather than by individual problems with resilience or coping, and thus changes in the work environment are key to reducing risk (5). In this sense, psychosocial factors have been found potential risk factors for various health outcomes for the general working population and for healthcare professionals (8). It can be positive and influence the wellbeing of the workers, but that can also produce stress and negatively impact on health perception (8).

Two widely recognized theoretical models the Job Demand-Control-Social support (JDC) and the Effort-Reward Imbalance (ERI) model, provide robust frameworks for understanding how specific workplace stressors contribute to burnout (9, 10). The JDC model posits that high job demands coupled with low decision latitude increase the risk of psychological strain and emotional exhaustion. In contrast, the ERI model emphasizes the imbalance between the effort invested and the rewards received (e.g., salary, recognition, career opportunities), suggesting that such discrepancies can lead to sustained stress and burnout. Empirical studies have validated these models in healthcare settings, showing that high demands, low control, and insufficient rewards are significantly associated with increased burnout symptoms and end up affecting health among medical professionals (11).

A meta-analysis that included studies of burnout in residents in different countries and continents, before de COVID-19 pandemic, showed a pooled overall prevalence of burnout of 27.7% in European countries (12). A meta-analysis of burnout studies in our country observed a prevalence of 24% in physicians including residents (13).

The outbreak of the COVID-19 pandemic at the end of 2019 represented a health emergency throughout the world and caused a situation of major stress for health personnel (14). Consequences for the mental health and recovery of healthcare workers treating and caring for patients with COVID-19 has been an important public health problem that remains in some cases (15). However, the changes that the pandemic has produced in psychosocial work factors and health, and the repercussion in health organizations and workers are not completely clarified.

The aims of this survey were to estimate the prevalence of burnout and the perceived health in medical residents after the end of pandemic, and the psychosocial risk factors, stress profile and overall job satisfaction associated with both. Moreover, we contrasted our results with a previous survey done in 2018, using the same methodology, to explore the variation of psychosocial risk factors, health perception, stress profile and overall job satisfaction of medical residents after the end of the pandemic period.

## 2 Material and methods

### 2.1 Design and participants

This was an observational cross-sectional study in a teaching hospital. Data were collected retrospectively from the results of a previous survey, through an online, voluntary, an anonymous questionnaire, through the validated FPSICO 4.0 online questionnaire (16), sent to all residents after the COVID-19 pandemic (2023). It was part of the collective health surveillance of health resident at the hospital. Data was also compared with a previous online survey sent to all residents before pandemic (2018) (17). Data from 2023 and 2018 surveys were independent samples.

The national specialized healthcare training program involves a progressive increase in responsibility, autonomy, and salary from the first year to the last ones. It lasts 4 to 5 years, depending on the chosen specialty. Most surgical specialties and some medical specialties (i.e. Internal Medicine) last 5 years, while the rest of specialties last 4 years. All residents do on-call shifts throughout the entire training period, with the exception of residents in Preventive Medicine, Dermatology and Occupational Health, who only do on-call shifts during the first two years of residency (18).

Our institution is a general teaching hospital with 800 beds, in an area of 540,000 inhabitants in a European city (>1,000,000 inhabitants). The whole number of residents in training in 2023 was 453. According to this data, and the prevalence of burnout in residents described in the literature (12, 13) the sample size required to estimate a proportion with an error of ± 5% and 95% confidence intervals (CI) under the assumption of uncertainty (p=0.30; q=0.70) would be 189 [180_199] responders to the survey. The Ethics Research Committee of the Institution approved the study (HCB/2023/0590).

### 2.2 Instruments and measures

The online survey questionnaire was sent to all residents (N = 453) without any exclusion criteria, and only included the data of those who answered all items (without missing), it included: a) Socio-demographic characteristics, gender, year of residency, from first year (R1) to fourth/fifth year (R4/5), type of specialty (medical, surgical and transversal), and if the resident does shifts. b) The Psychosocial factors questionnaire (F-PSICO) (19) comprises 89 closed questions related to nine psychosocial factors (17 subscales): Working time: it refers to rest periods during the working day and the impact of time dedicated to work on social and personal life (conciliation). Autonomy: the individual capacity for decision-making both in matters of working time and in the organization of work. Workload: the work demand that the worker faces. It assesses time pressure, effort of attention, and the amount and difficulty of the task. Psychological demands: the cognitive and emotional demands of the task. Variety of content: the extent to which work contains varied and meaningful task being performed. Participation/supervision: the worker´s perception of his or her possibilities of participation in different aspects of the job, and the perception of the control that the organization exercises over the supervision of his tasks. Worker interest/compensation: the degree of concern of the company for the worker regarding promotion, training or professional development; perception of job security, salary; balance between the effort made by the worker and the reward he obtains. Role performance: it refers to clarity of role (definition of functions and responsibilities) and conflict of role (incongruent, contradictory demands or that represent an ethical conflict for the worker). Lastly, relationships and social support: quality of interpersonal relationships and exposure to conflict situations at work, violence or discrimination, and support from bosses and co-workers, understood as a positive stress-compensating factor. Each subscale is assessed using a 5-Likert scale. The F-PSICO questionnaire has shown good psychometric properties for assessing an organization’s psychosocial conditions based on the workers’ perceptions of different aspects of their work (19). c) The nine questions related to risk of burnout of the validated Short burnout questionnaire (CBB, 1997) (20), that assess the three dimensions of burnout proposed by Maslach (5) exhaustion, depersonalization and personal realization, using a 5-Likert scale (1=absent to 5=extremely). This questionnaire scores the global risk of burnout as low (9, 10), moderate (11–25) and high risk (24, 26–44) taking into account the three dimensions. Those with high-risk scores were considered as having burnout. d) The perceived health (general, mental and vitality scales) of the Spanish validated Short Form Survey questionnaire (SF-36) (21). The higher the score the better the health perception. e) The validated Stress profile questionnaire (22), that evaluates behavior (i.e. irritability, social withdrawal, changes in eating habits, sleep disturbances, increased use of alcohol, drugs, or cigarettes), somatic (i.e. headaches or migraines, muscle tension or pain, digestive issues, fatigue or low energy, rapid heartbeat, or sweating or cold hands), and cognitive (i.e. racing thoughts, memory problems, constant worrying or overthinking, negative thinking or pessimism) stress symptoms. Higher score implies greater perception of related symptomatology. f) Finally, one-closed question to evaluated overall job satisfaction.

### 2.3 Statistical analysis

Categorical variables (e.g., gender, year of residency, type of specialty, and hospital shift status) are presented as frequencies and percentages. All scores from the questionnaires —including psychosocial factors, perceived health (general health, mental health, and vitality), stress symptomatology (behavior, somatic, and cognitive dimensions), and overall job satisfaction— were transformed to a standardized 0–100 scale, where higher values indicate greater levels of the corresponding variable. These scores are reported as medians and interquartile ranges, due to their non-normal distributions.

Univariate comparisons between residents with and without burnout were conducted using the chi-squared test for categorical variables. The Mann–Whitney–Wilcoxon test was applied for comparisons between men and women, and between residents working shifts vs. not, with respect to perceived general and mental health, and vitality scores. For comparisons across more than two groups (year of residency and specialty), the Kruskal–Wallis test was applied.

Spearman’s rank correlation coefficient (ρ) was used to assess the strength and direction of the associations between psychosocial factors, stress dimensions, and job satisfaction (as independent variables), and perceived health outcomes (general health, mental health, and vitality, as dependent variables). Correlation strength was interpreted based on the absolute value of the coefficients as follows: |ρ| = 0.10–0.29=weak; 0.30–0.49=moderate; ≥0.50=strong. Negative coefficients indicate inverse associations, while positive coefficients reflect direct associations.

To identify factors associated with burnout outcome, a multivariate logistic regression model was constructed. First, a best subset selection procedure was applied to the psychosocial factors, stress dimensions, and job satisfaction, to identify the subset of variables that were independently and significantly associated with burnout. In the second step, resident characteristics (gender, type of specialty, and year of residency) were evaluated for inclusion and retained in the model if they showed a statistically significant association with burnout. The final model included questionnaire scores and relevant resident characteristics. Results are reported as adjusted odds ratios with 95% confidence intervals (CIs). Continuous predictors were scaled in 5-unit increments to improve interpretability.

Separate multiple linear regression models were developed to identify factors associated with each perceived health outcome (general health, mental health, and vitality), as measured by the SF-36. The variable selection procedure was the same as before: best subset selection was applied to psychosocial factors, stress dimensions, and job satisfaction, and resident characteristics were evaluated for inclusion and retained in the model if they showed a statistically significant association. Model results are presented as estimated regression coefficients, standard errors, and 95% CIs. In addition, the adjusted coefficient of determination (R²) is reported.

To examine changes in psychosocial risk factors, stress profiles, perceived health, and job satisfaction between the pre-pandemic (2018) and post-pandemic (2023) periods, linear regression models were used. Mean differences between the two time points were estimated along with 95% CIs, adjusting for gender and year of residency as covariates. These adjusted mean differences were interpreted as effect sizes to quantify the magnitude of change over time. In addition, for the purpose of comparability across scores, unadjusted standardized mean differences between pre- and post-pandemic scores were also computed.

Analyses were performed with the statistical software R (version 4.4.2) (Vienna, Austria; https://www.r-project.org/). Statistical significance was set at p < 0.05.

## 3 Results

### 3.1 Characteristics of the sample

We collected the answers of 43.5% of residents, a sample with higher proportion of women (64.0%), and similar participation by year of residency: R1 23.9%), R2 (25.9%), R3 (24.4%), and R4/R5 (25.9%). The 56.9% of residents were doing a medical specialty, 19.8% a surgical specialty, and 23.4% were doing a transversal medical specialty (i.e. radiology, laboratory…), and most of them (92.9%) were doing shifts at the hospital. See **Table 1**.

**Table 1:** Characteristics of the sample and burnout prevalence by subgroups.

Variables	All residents	Burnout +	Burnout –	x^2^	df	p
All	N=197 (100%)	N=55 (27.9%)	N=142 (72.1%)			
Gender				0.011	1	0.915
-Women	126 (64.0%)	36 (28.6%)	90 (71.4%)			
-Men	71 (36.0%)	19 (26.8%)	52 (73.2%)			
Year of residency				8.276	3	0.041
-R1	47 (23.9%)	6 (12.8%)	41 (87.2%)			
-R2	51 (25.9%)	14 (27.5%)	37 (72.5%)			
-R3	48 (24.4%)	16 (33.3%)	32 (66.7%)			
-R4/R5	51 (25.9%)	19 (37.3%)	32 (62.7%)			
Type of specialty				8.126	2	0.017
-Medical	112 (56.9%)	27 (24.1%)	85 (75.9%)			
-Surgical	39 (19.8%)	18 (46.2%)	21 (53.8%)			
-Transversal	46 (23.4%)	10 (21.7%)	36 (78.3%)			
Doing shifts				0.758	1	0.384
-Yes	183 (92.9%)	53 (29.0%)	130 (71.0%)			
-No	14 (7.1%)	2 (14.3%)	12 (85.7%)			

R1= First year of residency; R2= Second year of residency; R3=Third year of residency; R4/R5= Fourth/fifth year of residency.

p-value is obtained from chi-squared test.

### 3.2 Burnout

The prevalence of burnout in the residents after the COVID-19 pandemic was 27.9%. There were not significant differences between women and men (p=0.915). Burnout prevalence increased with years of residency from 12.8% in the first year to 37.3% in the last year (p=0.041). Burnout prevalence was higher in those residents training in a surgical specialty (46.2%) compared with those in medical (24.1%) or transversal specialty (21.7%) (p=0.017). No statistically significant differences were observed between those residents doing or not doing shifts during the residency (p=0.384). See **Table 1**.

Related to psychosocial factors those residents with burnout presented worse time of work (50.0 vs. 66.7, p<0.001), a psychosocial risk factor related to the balance of personal and work life, less temporary autonomy (25.0 vs. 41.7, p<0.001), and capacity of take decisions (33.3 vs. 42.9, p=0.005). On the other hand, burnout residents perceived more time pressure (66.7 vs. 55.6, p<0.001), effort of attention (68.4 vs. 63.2, p<0.001), and more quantity and difficulty at work (69.2 vs. 57.7, p<0.001), with more cognitive (80.0 vs. 73.3, p=0.004) and psychological demands (72.0 vs. 52.0, p<0.001). Moreover, they perceived less variety sense of work (69.6 vs. 78.3, p<0.001), less participation (19.0 vs. 28.6, p=0.026) and supervision (50.0 vs. 66.7, p=0.002), less information/training/promotion (50.0 vs. 62.5, p<0.031), and less compensation (33.3 vs. 50.0, p<0.001) than those residents without burnout. Related to the perceived role performance, residents with burnout perceived more role conflicts (46.7 vs. 26.7, p<0.001) and less clarity of role (44.4 vs. 61.1, p<0.001) than those without burnout. Finally, those with burnout perceived less social support (63.2 vs. 63.7, p=0.006) and less interpersonal relationship, although this last psychosocial factor was not statistically significant comparing with residents without burnout (41.7 vs. 50.0; p>0.05). See **Supplementary Table 1**.

Burnout residents compared with those without burnout showed higher scores of stress behavior, somatic and cognitive symptoms (62.5 vs. 37.5, p<0.001; 37.5 vs. 12.5, p<0.001; and 50.0 vs. 25.0, p<0.001, respectively). Finally, residents with burnout had a statistically significant worse overall job satisfaction than those without burnout (33.3 vs. 66.7, p<0.001). See **Supplementary Table 1**.

The results of the multivariate logistic regression model examining factors associated with burnout among residents are presented in **Table 2**. The model was constructed using a best subset selection approach, as multiple psychosocial scores were significantly associated with burnout in preliminary analyses. Five variables remained independently associated with burnout in the final model. Higher levels of role conflict [adjusted odds ratio associated with a 5-unit increase (aOR)= 1.19; 95%CI= 1.05 – 1.36] and behavior stress symptoms (aOR= 1.24; 1.06 – 1.43) were linked to increased odds of burnout, while greater role clarity (aOR= 0.8; 0.68 – 0.94) and lower overall job satisfaction (aOR= 0.77; 0.67 – 0.90) were associated with lower odds. Year of residency also showed a significant association, with more advanced residents (R4/R5) showing substantially higher odds of burnout (aOR= 8.95; 2.02-39.66) compared to first-year residents.

**Table 2:** Estimated odds ratio for burnout syndrome obtained from logistic regression mode.

Outcome	Predictor variables	Adjusted OR	95% CI	p-value^1^
Burnout	Conflict of role^2^	1.19	1.05 – 1.36	0.006
	Clarity of role^2^	0.80	0.68 – 0.94	0.006
	Behavior stress^2^	1.24	1.06 – 1.43	0.004
	Overall job satisfaction^2^	0.77	0.67 – 0.90	<0.001
	Year of residence: (Ref: R1)			0.019
	R2	4.33	1.00 – 18.72	
	R3	4.29	0.95 – 19.28	
	R4/R5	8.95	2.02 – 39.66	

^1^ Obtained from likelihood ratio test.

^2^ Odds ratio associated with 5-unit increase.

### 3.3 Perceived general health, mental health and vitality

Women perceived a slightly worse general (65.0 vs. 60.0) and mental health (60.0 vs. 64.0) without statistical significant (p>0.05, respectively) and less vitality (45.0 vs. 50.0, p=0.022) during the residency. There were not statistical differences (p>0.05) related to year of residency, type of specialty or doing shifts. See **Table 3**.

**Table 3:** Perceived general and mental health, and vitality mean scores by gender, year of residency, type of specialty and doing shift.

Variables	General health		Mental health		Vitality	
	Median [Q1-Q3] ^1^	p-value^2^	Median [Q1-Q3] ^1^	p-value^2^	Median [Q1-Q3] ^1^	p-value^2^
Gender		0.254		0.163		0.022
-Women	65.0 [52.5 – 77.5]		60.0 [44.0 – 72.0]		45.0 [30.0 – 55.0]	
-Men	70.0 [57.5 – 80.0]		64.0 [50.0 – 76.0]		50.0 [40.0 – 60.0]	
Year of residency		0.895		0.574		0.872
-R1	65.0 [52.5 – 77.5]		64.0 [48.0 – 80.0]		50.0 [32.5 – 60.0]	
-R2	72.0 [57.5 – 77.5]		60.0 [48.0 – 72.0]		50.0 [35.0 – 55.0]	
-R3	66.2 [52.5 – 77.5]		60.0 [40.0 – 76.0]		45.0 [30.0 – 60.0]	
-R4/R5	60.0 [55.0 – 75.0]		60.0 [44.0 – 76.0]		45.0 [27.5 – 60.0]	
Type of specialty		0.308		0.497		0.701
-Medical	66.2 [23.1 – 77.5]		60.0 [28.0 – 76.0]		45.0 [30.0 – 60.0]	
-Surgical	60.0 [18.8 – 73.8]		60.0 [32.0 – 72.0]		45.0 [22.5 – 52.5]	
-Transversal	73.8 [25.0 – 80.0]		64.0 [19.0 – 71.0]		50.0 [18.8 – 55.0]	
Doing shifts		0.955		0.977		0.607
-Yes	67.5 [22.5 – 77.5]		60.0 [28.0 – 76.0]		45.0 [30.0 – 60.0]	
-No	68.8 [21.2 – 77.5]		62.0 [28.0 – 75.0]		42.5 [33.8 – 60.0]	

^1^ Q1: first quartile; Q3: third quartile.

^2^ Obtained from either Wilcoxon or Kruskal-Wallis test.

**Supplementary Table 2** shows the strength of relationship between perceived health, and psychosocial factors, stress profile and overall job satisfaction using Spearman´ correlation coefficient. General health correlated moderately and negatively with role of conflict (r = -0.368; p<0.001), and strongly and negatively with somatic stress (r = -0.503; p<0.001), behavior stress (r = -0.529; p<0.001), and cognitive stress (r = -0.517; p<0.001); and moderately and positively with overall job satisfaction (r = 0.357; p<0.001). Mental health correlated moderately and positively with time of work (r = 0.3; p<0.001), temporary autonomy (r = 0.323; p<0.001), variety sense of work (r = 0.344; p<0.001), supervision (r = 0.311; p<0.001), compensation (r = 0.308; p<0.001), and clarity of role (r = 0.354; p<0.001); moderately and negatively with time pressure (r = -0.343; p<0.001), effort of attention (r = -0.354; p<0.001), quantity and difficult of work (r = -0.382; p<0.001) and conflict of role (r = -0.408; p<0.001); strongly and negatively with behavior stress (r = -0.724; p<0.001), somatic stress (r = -0.59; p<0.001), and cognitive stress (r = -0.62; p<0.001); and moderately and positively with overall job satisfaction (r = 0.47; p<0.001). Vitality correlated moderately and positively with temporary autonomy (r = 0.309; p<0.001); strongly and negatively with role conflict (r = -0.45; p<0.001); moderately and negatively with time pressure (r = -0.348; p<0.001), effort of attention (r = -0.354; p<0.001), and quantity, difficulty of work (r = -0.383; p<0.001; strongly and negatively with behavior stress (r = -0.664; p<0.001), somatic stress (r = -0.543; p<0.001) and cognitive stress (r = -0.583; p<0.001); and moderately and positively with overall job satisfaction (r = 0.469; p<0.001).

**Table 4** exposes multivariate linear regression final models of perceived health outcomes. In the perceived general health outcome showed that the variables that remained statistically significant in the final model were somatic and cognitive stress, supervision and conflict of role, showing that residents with low somatic and cognitive stress, low conflict of role, and high supervision show better perceived general health. These variables explained 35% of the variation in perceived general health (adjusted-R^2^ = 0.353). Regarding perceived mental health the final model indicated that lower levels of behavior and somatic stress, reduced information/training/promotion, higher temporary autonomy, effective supervision, and greater overall job satisfaction were associated with higher perceived mental health scores. Together, these variables accounted for 68% of the variance in perceived mental health (adjusted R²=0.683). Concerning perceived vitality the final model shower that higher perceived vitality was associated with a lower stress profile (behavior, somatic, and cognitive), lower role conflict, greater decision-making, autonomy, and being male. These variables explained 62% of the variance in perceived vitality scores (adjusted R²=0.624).

**Table 4:** Multiple linear regression models of perceived health (SF-36) (general health, mental health and vitality subscales).

General Health subscale (SF-36)	B	SE	95%CI	p-value
(Constant)	73.92	4.43		
1. Supervision	0.17	0.06	[0.05, 0.29]	0.005
2. Conflict of role	-0.19	0.05	[-0.30, -0.07]	0.002
3. Somatic stress	-0.18	0.07	[-0.32, -0.03]	0.015
4. Cognitive stress	-0.21	0.06	[-0.33, -0.09]	0.001
Adjusted R^2^	0.353			
Excluded variables: Year of residency, gender, and type of specialty; Time of work; Temporary autonomy; Take decisions; Time pressure; Effort of attention; Quantity, difficulty of work; Emotional demands; Variety sense of work; Participation; Compensation; Clarity of role; and Social Support; Behavior stress; and Overall job satisfaction.				
Mental Health subscale (SF-36)	B	SE	95%CI	p-value
(Constant)	74.32	5.38		
1. Temporary autonomy	0.10	0.03	[0.03, 0.16]	0.005
2. Supervision	0.13	0.05	[0.03, 0.23]	0.011
3. Information/training/promotion	-0.07	0.03	[-0.14, -0.01]	0.022
4. Behavior stress	-0.52	0.06	[-0.63, -0.41]	< 0.001
5. Somatic stress	-0.24	0.06	[-0.35, -0.13]	< 0.001
6. Overall job satisfaction	0.12	0.05	[0.01, 0.22]	0.029
Adjusted R^2^	0.683			
Excluded variables: Year of residency, gender, and type of specialty; Time of work; Take decisions; Time pressure; Effort of attention; Cognitive demands; Emotional demands; Participation; Compensation; Clarity of role; Conflict of role; and Social Support; and Cognitive stress.				
Vitality subscale (SF-36)	B	SE	95%CI	p-value
(Intercept)	75.78	3.99		
1. Take decisions	0.11	0.05	[0.01, 0.21]	0.030
2. Conflict of role	-0.18	0.05	[-0.29, -0.07]	< 0.001
3. Behavior stress	-0.33	0.07	[-0.47, -0.19]	< 0.001
4. Somatic stress	-0.19	0.07	[-0.32, -0.06]	0.005
5. Cognitive stress	-0.18	0.06	[-0.29, -0.06]	0.003
6. Gender*	-5.99	1.97	[-9.88, -2.11]	0.003
Adjusted R^2^	0.624			
Excluded variables: Year of residency and type of specialty; Work time; Temporary autonomy; Time pressure; Effort of attention; Quantity, difficulty of work; Cognitive demands; Emotional Demands; Variety sense of work; Participations; Supervision; Information/training/promotion; Compensation; Clarity of role; and Social Support; and Overall job satisfaction.				

*Reference: women.

### 3.4 Changes pre/post-pandemic period related to psychosocial factors, stress profile and perceived health and overall job satisfaction.

**Figure 1:**
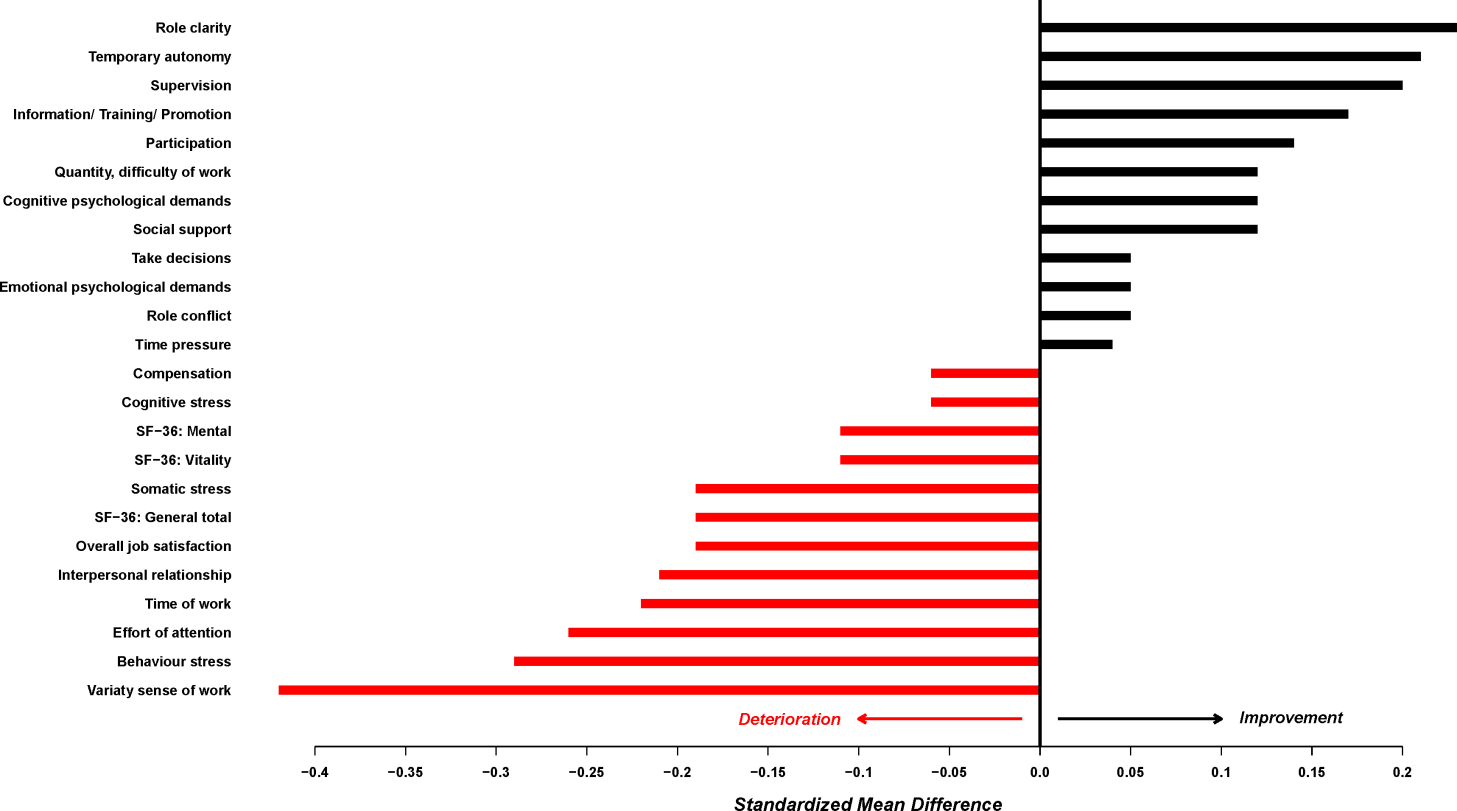
Unadjusted standardized mean differences between psychosocial factors, stress profile and perceived health (SF-36) between residents pre-pandemic (2018) and post-pandemic (2023).

We compared the present results with those of a previous survey done in 2018 in an independent sample of residents in the same teaching hospital, answered by 107 (31%) residents (12). **Figure 1** shows the unadjusted standardized mean differences of psychosocial factors, stress profile, perceived health (SF-36), and overall satisfaction between residents´ pre-pandemic (2018) and post-pandemic (2023) scores. Considering the direction of the variables we organized them in two groups: those with deterioration and those with improvement after the COVID-19 pandemic. On one hand, crude results underline the deterioration of varied sense of work and effort of attention psychosocial factors, and behavior stress; and on the other, the improvement of clarity of role, temporary autonomy, and supervision psychosocial factors, although without reaching statistical significance level.

Finally, we present the mean differences adjusted by gender and year (ΔMean) of residency (the two socio-demographic characteristics present in both surveys, 2023 and 2018). See **Supplementary Table 3**. Three variables –varied sense of work (ΔMean=-5.7, 95%CI: -8.8 – -2.5; p<0.001), effort of attention (ΔMean=4.5, 95%CI: 0.6 – 8.4; p=0.026), and behavior stress (ΔMean=5.5, 95%CI: 0.8 – 10.1; p=0.022) – showed statistically significant deterioration; and temporary autonomy (ΔMean=4.5, 95%CI: 0.1 – 11.4; p=0.045) presented a statistically significant improvement. Overall job satisfaction was not statistically significant different between pre/post-pandemic periods.

## 4 Discussion

The main results of the study showed that one in three residents had burnout by the end of the pandemic. The prevalence of burnout increased over the course of residency, and was higher among those training in surgical specialties. Role performance of residents was a key psychosocial risk factor for burnout, together with behavior stress symptoms. Burnout was associated with low overall job satisfaction. Moreover, the study highlights the importance of conflict of role, supervision, temporary autonomy, and the possibility of take decisions psychosocial risk factors affecting residents’ perceived health. Stress symptoms (behavior, somatic and cognitive) were also associated with worse perceived health, as was female gender. Finally, when comparing the pre/post-pandemic period, we found that residents perceived a significant deterioration of the psychosocial factors variety sense of work, effort of attention, and greater behavior stress levels, alongside with an improvement in temporary autonomy.

The prevalence of burnout in medical residents found in our study shows worrying figures, but within the ranges described both before and during the first and third wave of the pandemic (12, 13, 23). This finding indicates that burnout in healthcare workers as is the case of residents, is related to a chronically stressful work environment, and is not primarily related to current or past health emergencies. In such situations, indirect exposure to traumatic events can more frequently trigger other conditions in the worker, such as secondary post-traumatic stress syndrome (24). Longer-term assessments of post-pandemic burnout remain necessary. Consistent with the systematics reviews of burnout pre-pandemic prevalence, we found no significant gender differences in burnout rates and surgical specialties showed higher rates as pre-pandemic time (12, 25–28). Burnout worsened throughout residency from the first to last year, being the higher prevalence in the last years of training, independent of other predictor variables, as shown by adjusted odds ratios. However, we observed an increase of burnout prevalence in the second year higher than 50%, suggesting the need to implement early prevention measures to avoid burnout development. Second year of training requires more clinical and academic responsibilities, and residents should to start on-calls of their specialty, perceiving probably not enough supervision (18). This is in concordance with the results of a previous pandemic study. In this study, we pointed out the profile of risk of psychosocial factors of this period of training (second year of residency), such as workload, cognitive and psychological demands and supervision (17).

The burnout findings are consistent with the theoretical models previously described, according to which specific psychosocial factors in the work environment and low job satisfaction, constitute key determinants in the onset of burnout (9, 10). In this sense, burnout showed a statistical significant association with all psychosocial risk factors, except interpersonal relationship, together with high levels of stress symptomatology and low overall job satisfaction. However, role performance was the psychosocial risk factor most strongly associated with post-pandemic burnout in our residents. While pre-pandemic studies had linked this factor to burnout (29), the pandemic’s chaotic context exacerbated role ambiguity and conflict (30). Some of the changes that the pandemic produced in resident’s work role could continue in many cases after the end of the pandemic implying the maintenance of a role ambiguity and the repercussion on present burnout.

Better perceived general health after pandemic was associated with less conflict of role and more supervision, both psychosocial risk factors, and less perceived somatic and cognitive stress. The balance between supervision and independence is difficult to achieve in daily practice, and changes during pandemic period may have led to a greater imbalance in resident supervision once finished (31). This may mean that on many occasions supervision will need to be adjusted to each resident, readjusted at different point in time along the residency, and be based on a relationship of mutual trust between supervisor and resident (32). A better perceived mental health was also associated with supervision and temporary autonomy. These results indicate the convenience of jointly improving these two psychosocial risk factors during the residency. Interestingly, information/training/promotion activities showed an inverse correlation with mental health, which might suggest that a reduced training load (e.g., preparing fewer clinical sessions, attending fewer conferences…) could have led to decreased resident stress associated with teaching and research demands, ultimately improving perceived mental health (30, 33). Perceived vitality was most strongly associated with conflict of role. Greater conflict of role was a psychosocial risk factor that also correlated with other outcomes such as worse perceived general health, and higher burnout risk. Pandemic had induced role ambiguity generating anxiety, harming health and energy levels (15), which could affect health in the post-pandemic period. The result reinforces the importance of avoid incongruent or contradictory demands for the resident, and adequately defining the resident’s role to elude repercussions on their health. Take decision—tied to temporary autonomy—also explained better vitality, and that was favored during the pandemic. Similar to pre-pandemic time (17), gender also was associated with vitality, which was lower in women probably correlated with gender and socio-cultural determinants (34).

The impact of stress on perceived health of young physicians in training has been well documented in the literature (35). Both burnout and poor self-perceived health were associated with stress-related symptoms in our study, especially behavior manifestations such as irritability, difficulty relaxing, avoidance behaviors, and disruptions in sleep, eating patterns and substance use (4). Early and structured stress management interventions such psychological support, relaxation training, and healthy lifestyle promotion, may be useful to protect the general and mental health of medical trainees and improve outcomes for the overall healthcare system (36).

The comparison of pre/post-pandemic psychosocial factors in residents revealed significant deterioration in variety sense of work and effort of attention, alongside improved temporary autonomy. These findings are in line with the disruptive effects of COVID-19 on residency training and healthcare organization (30). Particularly noteworthy is the near-significant effect of time of work, reflecting the critical work-life balance challenges faced by healthcare professionals who endured extended shifts and were frequently required to quarantine (24). Related to pre-pandemic period we observed a deterioration of behavior stress symptoms, suggesting that the stress generated during the pandemic continues to play a role in health after it ends. On respect to resident perception of the improved temporary autonomy this psychosocial factor showed the greater magnitude of change, suggesting that residents gained some level of control over how they get work done related to pre-pandemic period. We found that temporary autonomy was also associated with a better perception of mental health after pandemic. Other studies support these results, showing perceived autonomy associated with wellbeing and more job satisfaction, meanwhile perceived low autonomy has been associated with burnout (37, 38).

The study is not without limitations. This was a retrospective cross-sectional study, which precludes causal inference (39). Since this was a single-hospital study, the results may not be generalizable to other institutions. The pre/post pandemic analysis compared two different independent samples due to the complete resident turnover each four/five years. Moreover, due to the participation rate of the study, mainly the pre-pandemic survey, we cannot exclude a potential selection bias concern (40). Physicians as a group tend to show lower response rates than other professional groups (41). Web-based surveys have generally lower response rates than face to face or telephone interviews or mail surveys (42). The online survey had not collect information about having had post-COVID-19 condition (43). Regarding the logistic regression model, we acknowledge the relatively wide confidence intervals for the residency year categories (R2, R3, and R4/R5). This reduced precision stems from the small number of burnout cases within the R1 reference group (n=6). Nevertheless, the adjusted point estimates remained directionally consistent with the unadjusted odds ratios, and the continuous predictors demonstrated high stability and narrow confidence intervals, suggesting that the primary model findings remain robust. Finally, while COVID-19 was a clear disruptor in work setting (30) and in general in the society (44, 45), other factors may have influenced observed differences.

## 5 Conclusions

Future reassessments will be necessary to determine whether the observed patterns persist under different epidemiological conditions. The pandemic forced widespread adaptations to our work modalities, with many of these changes having significant health impacts. Several modifications - such as teleworking and virtual meetings - have become permanent features of professional life (46). It would be interesting long-term studies to evaluate the sustained effects on health outcomes. Although burnout rates of medical residents did not rise post-pandemic, the levels are still worrying, so it is necessary to implement prevention plans according to each organization. To do this, it is important to know the current psychosocial risk factors, as well as the impact on health. Our study emphasizes role performance as the main psychosocial risk factor, and high levels of stress, especially behavior symptomatology after the pandemic, making it mandatory to implement preventive interventions and health promotion plans from early time in the residency. On one hand, it would be crucial to guarantee compliance with the responsibilities assigned in the training itinerary, establishing a clear delineation between the functions of residents and the medical assistant, and providing clear and reliable channels to manage the situations in which the established role definition is not met. On the other hand, it would be important to enhance progressive temporary autonomy to the trainee along the years of residency (37, 38). As well as to implement health promotion plans of residents based on the maintenance of wellbeing, such as sleep quality, healthy eating, physical activity and training for good stress management.

## Data Availability

The data analyzed in this study is subject to the following licenses/restrictions: Data base are available from authors under request. Requests to access these datasets should be directed to Rocío Martin-Santos, rmslaffon@gmail.com.
