# Supplementary material for: Burnout and perceived health in medical residents after the COVID-19 pandemic: a single-center cross-sectional study
Source: Front Psychiatry. 2026 Jun 19;17:1659089. doi: 10.3389/fpsyt.2026.1659089 (PMC13328093; doi:10.3389/fpsyt.2026.1659089)
Supplement: Supplementary file 1 [file Table1.pdf]

Supplementary Table 1. Univariate analysis of psychosocial factors and stress profile and overall job satisfaction related to burnout in residents

**Psychosocial factors**	**Burnout +** (N=55) **Median [Q1-Q3][1]**	**Burnout –** (N=142) **Median [Q1-Q3][1]**	p[2]
Time of work	50.0 [33.3 - 66.7]	66.7 [50.0 - 83.3]	<0.001
Temporary autonomy	25.0 [16.7 - 41.7]	41.7 [25.0 - 58.3]	<0.001
Take decisions	33.3 [23.8 - 42.9]	42.9 [28.6 - 52.4]	0.005
Time pressure	66.7 [66.7 - 88.9]	55.6 [44.4 - 75.0]	<0.001
Effort of attention	68.4 [63.2 - 84.2]	63.2 [52.6 - 68.4]	<0.001
Quantity, difficulty of work	69.2 [57.7 - 76.9]	57.7 [46.2 - 69.2]	<0.001
Cognitive demands	80.0 [63.3 - 93.3]	73.3 [60.0 - 86.7]	0.040
Psychological demands	72.0 [58.0 - 76.0]	52.0 [44.0 - 67.0]	<0.001
Variety sense of work	69.6 [60.9 - 78.3]	78.3 [69.6 - 87.0]	<0.001
Participation	19.0 [9.5 - 33.3]	28.6 [19.0 - 33.3]	0.026
Supervision	50.0 [33.3 - 66.7]	66.7 [50.0 - 66.7]	0.002
Information/training/promotion	50.0 [37.5 - 62.5]	62.5 [50.0 - 87.5]	0.031
Compensation	33.3 [25.0 - 50.0]	50.0 [41.7 - 66.7]	<0.001
Clarity of role	44.4 [33.3 - 55.6]	61.1 [50.0 - 66.7]	<0.001
Conflict of role	46.7 [33.3 - 60.0]	26.7 [13.3 - 33.3]	<0.001
Interpersonal relationship	41.7 [25.0 - 66.7]	50.0 [16.7 - 75.0]	0.816
Social support	63.2 [52.6 - 73.7]	73.7 [57.9 - 84.2]	0.006
**Stress profile**			
Behaviour symptoms	62.5 [50.0 - 71.9]	37.5 [25.0 - 50.0]	<0.001
Somatic symptoms	37.5 [21.9 - 53.1]	12.5 [6.3 - 25.0]	<0.001
Cognitive symptoms	50.0 [37.5 - 75.0]	25.0 [12.5 - 43.8]	<0.001
**Overall job satisfaction**	33.3 [33.3 - 66.7]	66.7 [66.7 - 66.7]	<0.001

[1] Q1: first quartile; Q3: third quartile
[2] Obtained from Wilcoxon test

Supplementary Table 2. Spearman correlations between psychosocial factors, stress profile and overall job satisfaction, and perceived general and mental health and vitality

**Psychosocial factors**	**General health**	**Mental health**	**Vitality**
Time of work	0.256**	0.300**	0.297**
Temporary autonomy	0.259**	0.323**	0.309**
Take decisions	0.136	0.275**	0.235**
Time pressure	-0.278**	-0.343**	-0.348**
Effort of attention	-0.187**	-0.354**	-0.337**
Quantity, difficulty of work	-0.260**	-0.382**	-0.383**
Cognitive demands	-0.140	-0.111	-0.165*
Psychological demands	-0.256**	-0.299**	-0.274**
Variety sense of work	0.247**	0.344**	0.300**
Participation	0.121	0.109	0.169*
Supervision	0.250**	0.311**	0.324**
Information/training/Promotion	0.092	0.135	0.204**
Compensation	0.285**	0.308**	0.415**
Clarity of role	0.292**	0.354**	0.326**
Conflict of role	-0.368**	-0.408**	-0.450**
Interpersonal relationship	0.073	0.020	-0.019
Social support	0.226**	0.284**	0.202**
**Stress profile**			
Behaviour symptoms	-0.529**	-0.724**	-0.664**
Somatic symptoms	-0.503**	-0.590**	-0.543**
Cognitive symptoms	-0.517**	-0.620**	-0.583**
**Overall job satisfaction**	0.357**	0.470**	0.469**

*p<0.01; **p<0.001

Supplementary Table 3. Changes in psychosocial factors, stress profile and perceived health and overall satisfaction between pre-pandemic and post-pandemic period.

Psychosocial factors	2018 Mean	2023 Mean	Δ Mean[1]	95% CI	p[2]
Time of work	52.7	54.4	4.9	[0.0, 9.9]	0.051
Temporary autonomy	36.5	41.5	5.8	[0.1, 11.4]	0.045
Take decisions	38.9	39.9	1.4	[- 3.1, 6.0]	0.528
Time pressure	64.2	63.2	- 0.7	[- 5.9, 4.5]	0.779
Effort of attention	58.9	63.3	4.5	[0.6, 8.4]	0.026
Quantity, difficulty of work	62.6	60.7	- 2.0	[- 5.7, 1.7]	0.265
Cognitive psychological demands	77.5	75.4	- 2.2	[- 6.2, 1.8]	0.281
Emotional psychological demands	58.2	57.2	- 1.0	[- 5.4, 3.5]	0.665
Variety sense of work	80.6	75.0	- 5.7	[- 8.8, - 2.5]	< 0.001
Participation	23.2	25.2	2.4	[- 1.1, 6.0]	0.170
Supervision	50.9	54.6	3.9	[- 0.5, 8.2]	0.081
Information/training/promotion	55.0	59.5	4.4	[- 2.0, 10.8]	0.180
Compensation	50.2	49.1	- 1.2	[- 5.8, 3.5]	0.622
Role clarity	51.3	55.1	3.8	[- 0.2, 7.7]	0.063
Role conflict	30.8	29.9	- 0.3	[- 4.8, 4.2]	0.896
Interpersonal relationship	48.4	42.3	- 6.2	[-13.0, 0.6]	0.075
Social support	68.7	70.7	2.0	[- 1.9, 5.9]	0.321
**Stress profile**					
Stress behaviour symptoms	38.5	44.4	5.5	[0.8, 10.2]	0.022
Stress somatic symptoms	20.2	23.7	2.8	[- 1.6, 7.2]	0.208
Stress cognitive symptoms	34.4	35.7	1.5	[- 4.0, 6.9]	0.589
**Perceived health (SF-36)**					
General	69.5	66.0	- 1.5	[- 7.4, 1.3]	0.173
Mental	61.4	59.3	- 1.3	[- 6.0, 3.1]	0.523
Vitality	47.6	45.6	- 3.2	[- 5.7, 3.1]	0.570
**Overall job satisfaction**	63.6	60.1	- 3.2	[- 7.5, 1.0]	0.135

[1]Standardized mean difference adjusted by gender and year of residency.

[2]Obtained from linear regression model adjusted by gender and year of residency
